# Qarles: a web server for the quick characterization of large sets of genes

**DOI:** 10.1093/nargab/lqaf030

**Published:** 2025-03-29

**Authors:** Carles Pons

**Affiliations:** Institute for Research in Biomedicine (IRB Barcelona), The Barcelona Institute for Science and Technology (BIST), 08028 Barcelona, Catalonia, Spain

## Abstract

The characterization of gene sets is a recurring task in computational biology. Identifying specific properties of a hit set compared to a reference set can reveal biological roles and mechanisms, and can lead to the prediction of new hits. However, collecting the features to evaluate can be time consuming, and implementing an informative but compact graphical representation of the multiple comparisons can be challenging, particularly for bench scientists. Here, I present Qarles (quick characterization of large sets of genes), a web server that annotates *Saccharomyces cerevisiae* gene sets by querying a database of 31 features widely used by the yeast community and that identifies their specific properties, providing publication-ready figures and reliable statistics. Qarles has a deliberately simple user interface with all the functionality in a single web page and a fast response time to facilitate adoption by the scientific community. Qarles provides a rich and compact graphical output, including up to five gene set comparisons across 31 features in a single dotplot, and interactive boxplots to enable the identification of outliers. Qarles can also predict new hit genes by using a random forest trained on the selected features. The web server is freely available at https://qarles.org.

## Introduction

Gene set characterization is a recurring task in computational biology in which properties of two gene sets are compared [1]. The initial gene annotation using features of interest is followed by statistical analyses comparing the values of both gene sets. This task is particularly relevant in large-scale experimental screens, which usually target a large fraction of the genome and identify a set of positive results, also known as hit genes [2, 3]. Characterizing the specific properties of the hit genes compared to the tested genes (i.e. background genes) can reveal their biological role and enable the prediction of hits among untested genes [4]. These properties can also provide details on the molecular mechanisms behind the biological process being studied [5].

However, selecting the gene features to analyze can be cumbersome, time consuming, and prone to selection bias. Thus, researchers characterizing gene sets often resort to popular databases like GO [6] or KEGG [7], which largely consist of literature-curated functional annotations and are biased toward the most studied genes [8]. An alternative is the evaluation of properties derived from high-throughput experiments, which are usually less biased [9], but collecting the relevant datasets may not be straightforward. Additionally, biological studies often test several conditions and/or perform different types of experiments [10], resulting in multiple lists of hit genes. The visual comparison of their specific properties can easily illustrate the distinct biological responses triggered. Still, selecting an optimal and informative way of presenting multiple enrichments in a single plot may prove challenging.

Thus, automation of the gene set characterization process is important to facilitate access to users lacking the needed expertise, but also to minimize errors, ensure reproducible results, and boost productivity. Online servers conveniently provide such automated solutions [1, 11]. To promote adoption by the scientific community, they should display a simple interface and a fast response time [11], and provide high-quality figures and reliable statistics.

Most of the servers, such as WebGestalt [12], g:Profiler [13], Flame [14], modEnrichr [15], Kobas [16], David [17], and Metascape [11], target several species, including *Saccharomyces cerevisiae*. However, these servers mostly focus their analyses on functional databases available for all targeted species, which largely consist of literature-curated annotations. Conversely, servers targeting only *S. cerevisiae* [18, 19] also evaluate features derived from high-throughput experiments, which can provide more mechanistic information. Even if few servers enable simultaneous analyses on several lists of genes [11], they all lack a global summarized visualization, and do not provide multiple gene set comparisons across several features in a single plot [11, 14, 19]. Moreover, in screens targeting only a fraction of the genome, the prediction of hits among the untested genes may be of interest. In such cases, relevant features of known hit genes can be used to train a prediction model and identify new hit genes [4]. However, servers performing enrichment analysis do not provide this functionality.

Here, I present Qarles, a free web server that identifies the specific properties of up to five sets of hit genes by querying a panel of 31 features used by the yeast community, enabling the prediction of new hits. Qarles provides rich and compact figures with a simple, intuitive, and quick interface, minimizing the entry barrier for nonexpert users.

## Materials and methods

### Interface

Qarles was implemented in R [20] using Shiny [21] and was designed to ensure all user interaction occurred within a single webpage for an efficient, seamless, and cohesive user experience. A minimal input form and a compact output enable an intuitive interface to navigate through the different results and functions provided by the server. The only required inputs are the ORFs of the hit and background genes, which should populate the “Hit list” and “Background list” boxes, respectively, and an identifier for the comparison. By default, all 31 available features are selected for analysis. A complete tutorial and detailed descriptions for the input, output, methodology, and the preloaded features are available on the website under the “Help”, “Features”, and “Tutorial” tabs. The “Release notes” tab will list any future changes in the methodology and/or features. Additionally, three use cases with real data illustrate how to use the tool. Importantly, no programming skills are required to use Qarles.

### Enrichments

**Figure 1.**
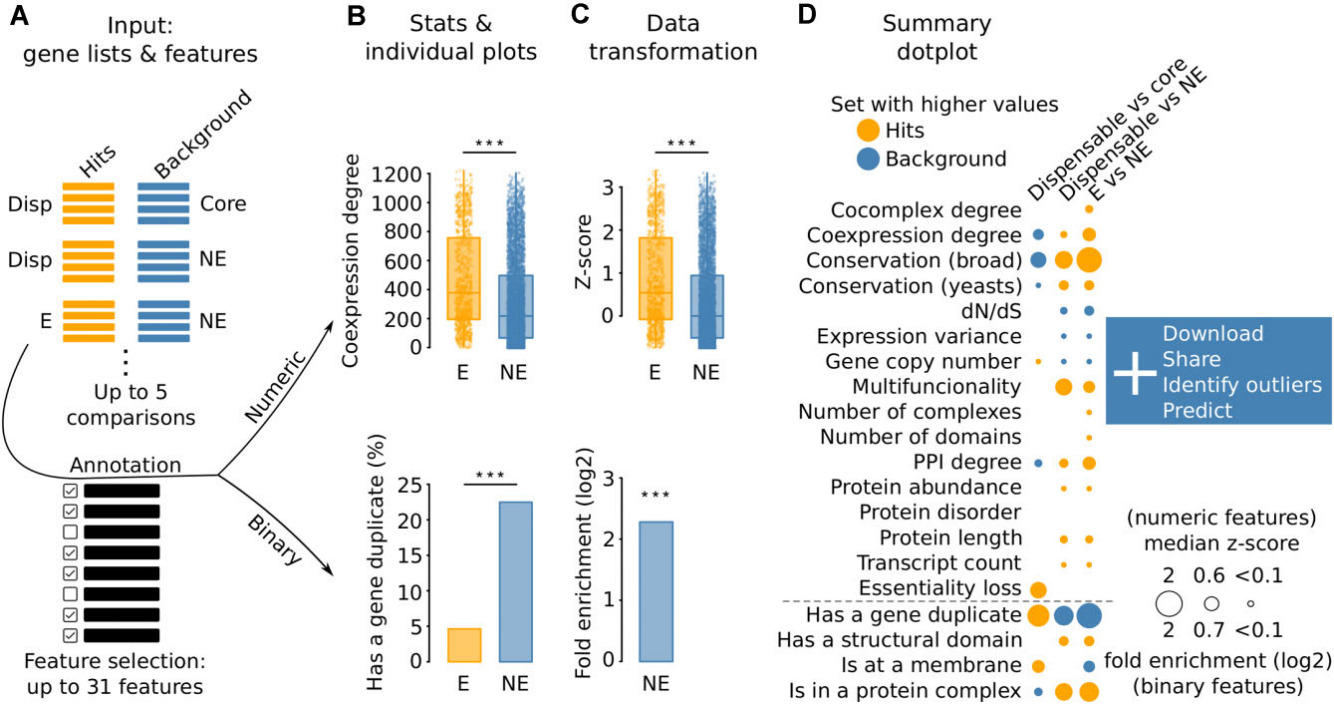
Qarles workflow example. (**A**) Dispensable essential genes (Disp, hits) are compared against core essential genes (Core, background) and nonessential genes (NE, background), and essential genes (E, hits) against nonessential genes (NE, background), across a subset of the 31 pre-selected features. (**B**) For numeric features (like coexpression degree, top), Qarles generates a boxplot with the corresponding values for E and NE, and calculates the statistical significance with a Mann–Whitney *U*-test. For binary features (like having a gene duplicate, bottom), Qarles generates a barplot with the corresponding ratios for E and NE, and calculates the statistical significance with a Fisher’s exact test. ***: *P* < .0005. (**C**) For numeric (top) and binary (bottom) features, Qarles summarizes the results using the median *z*-score value of the hits, and the log_2_ of the fold enrichment (see the “Materials and methods” section), respectively. ***: *P* < .0005. (**D**) Dotplot showing the summarized results for all gene set comparisons (Dispensable versus core, Dispensable versus NE, and E versus NE) across all the selected features. Dot color identifies the gene set (hits or background) with significantly higher values or ratios for numeric and binary features, respectively. Absent dots identify cases in which the difference is not significant. Finally, the user can download or share the results, identify outliers by accessing interactive plots, or predict new hit genes.

Qarles queries a panel of gene and protein features to quickly characterize the differences between a list of hit genes against a background set of genes. Users can compare up to five different hit and background gene sets across a panel of 31 features in a single analysis (Fig. 1A). Qarles processes numeric features (such as protein abundance or expression variance) and binary features (such as being part of a protein complex or being essential) differently (Fig. 1B).

For each numeric feature, Qarles performs a *z*-score normalization to the values of the hits by using the median and standard deviation of the values of the background genes (Fig. 1C, top). A resulting positive median *z*-score for the hits identifies a feature in which they tend to have higher values than the background genes. Conversely, a negative median *z*-score for the hits identifies a feature in which the background genes tend to have higher values. The statistical significance is calculated by means of Mann–Whitney *U*-tests. For each binary feature, Qarles calculates the fold enrichment as the ratio of hits with that particular feature divided by the equivalent ratio for the background genes (Fig. 1C, bottom). *P*-values are calculated with two-sided Fisher’s exact tests.

For each comparison between hit and background genes, users can correct for multiple tests by using the false discovery rate and Bonferroni methods.

### Features

Qarles includes 20 numeric and 11 binary pre-selected features that are popular among the yeast community. Numeric features include coexpression degree [22], conservation in yeast and distant species [23], dN/dS [23], expression variance upon environmental changes [24] and in different genetic backgrounds [25], gene copy number [23], messenger RNA (mRNA) half-life in CSM-lowURA media [26], multifunctionality [6], single mutant fitness [27], and transcript count [28]. Protein-level numeric features include co-complex degree [29], degradation rate [30], number of complexes [29], number of structural domains [31], PPI degree [23], abundance [32], disorder [33], half-life [30], and length [34].

Binary features include essentiality [4], having an unknown function [35], increasing lifespan upon deletion [36], being previously reported as acetylated, phosphorylated, or ubiquitinated in the literature [35], having a gene duplicate [35], and coding for a transcription factor [37], or a protein in a complex [29], with a structural domain [31], or localizing at a membrane [38].

Supplementary Table S1 in the supporting material and the “Features” tab in the website detail the data source for each feature, like the supplementary table or the download link and date. Features derived from data sources updated regularly, like co-complex degree or multifunctionality, which use data from the Complex Portal [29] and GO [6], respectively, will be recalculated once a year. Additionally, Qarles can easily incorporate other features upon popular demand, and users can also upload their own set of features to evaluate by clicking on the “Upload file” button next to the “Add features” label. Two example spreadsheets with user-defined features can be downloaded by clicking on “Example: Essentiality loss” and “Example: GI density.”

Importantly, understanding how the features were derived is essential for the biological interpretation of the results provided by Qarles. For instance, mRNA half-lives calculated in CSM-lowURA media [26] may not be relevant for hit genes identified in a different condition. Thus, users are encouraged to read the manuscripts describing the features and identify those relevant to their data.

### Prediction

Qarles uses the R library “randomForest” [39] to train a random forest model [40] with the features selected by the user for the prediction of novel hit genes. Importantly, only genes with valid data (i.e. different than “NA” values) for all features are considered by the predictor. The performance of the predictor on the training set (i.e. the hit and background lists) is calculated using the AUC (area under the curve) and reported to the user.

## Results and discussion

Qarles generates a graphical representation for each individual analysis, with boxplots for numeric features, and barplots for binary features (Fig. 1B). Additional interactive plots [41] can be accessed by clicking on the “Interactive plots” button to visually retrieve details on the statistical analyses and data quartiles, and to facilitate the identification of outliers.

Qarles conveniently provides a dotplot with a summarized global representation of the enriched features of each hit set (Fig. 1D). For each numeric feature, the dot size is proportional to the absolute median *z*-score normalized value of the hits (see the “Materials and methods” section), and the dot color identifies the gene set with higher feature values. For each binary feature, the dot size is proportional to the absolute value of the log_2_ of the fold enrichment (see the “Materials and methods” section), and the dot color identifies the gene set with a higher ratio for the particular feature. For both numeric and binary features, only dots representing significant differences are shown, after correcting for multiple tests with the method selected by the user. The dotplot can include up to five gene set comparisons across 31 features.

By clicking on “Download results”, users can retrieve a zip file containing the PDFs of the summary dotplot, and the individual bar charts and boxplots. In addition, the zip file contains spreadsheets with the gene values for the selected features and details on the statistical tests. The provided R script replicates the statistical tests and generates a basic version of the individual plots. By clicking on “Share your results”, users can also retrieve a URL to make their results available online. By pasting this link into a web browser, results can be conveniently accessed at a later time or easily shared with other researchers. The results will be available on the server at least for a month.

By clicking on “Predict hit genes”, Qarles trains a random forest for the prediction of hit genes and generates a downloadable table ranking the predictions for background (for potential retesting) and untested genes (i.e. *S. cerevisiae* genes not in the hit or background lists), with links to their corresponding *Saccharomyces* Genome Database (SGD) page [34]. The number of genes used for training and amenable to predictions are also reported.

Qarles was tested on Firefox and Chrome and the response time was under 15 s on the three provided online examples.

### Use cases

The website provides three examples to showcase the use of Qarles with real data, which illustrate the simplicity of the user interface and the quick response time in identifying significant enrichments that can be the starting point for predictions and downstream analyses.

**Figure 2.**
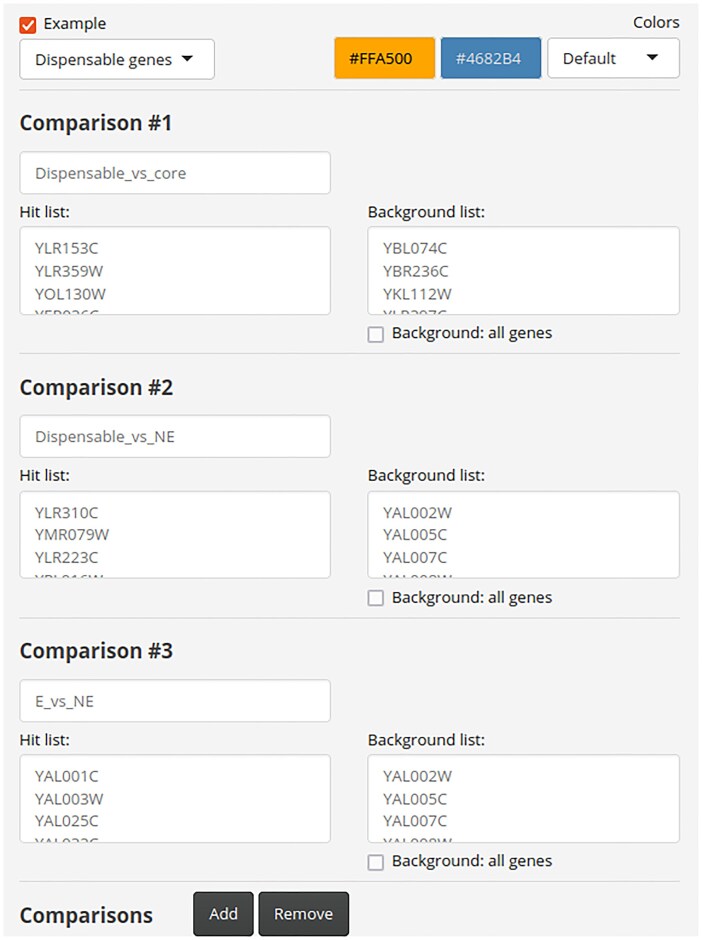
Qarles input. Screenshot of the input data forms using the example “Dispensable genes”, available on the website. Three different gene set comparisons are defined (Dispensable_vs_core, Dispensable_vs_NE, and E_vs_NE), and the “Hit list” and “Background list” boxes are populated with the corresponding gene sets.

The example labeled “Dispensable genes” replicates an analysis from a recent meta-study in which genes were classified by their essentiality [42]. By selecting this use case, three different gene set comparisons are defined (see Figs 1A and 2): in “Dispensable vs core”, the “Hit list” box is populated with dispensable essential genes (i.e. essential genes that become nonessential in the presence of suppressor mutations) and the “Background list” box with core essential genes (i.e. essential genes that are not dispensable), including data only from one study [4] to showcase the use of the predictor; in “Dispensable vs NE”, the “Hit list” and “Background list” boxes are populated with dispensable essential genes and nonessential genes, respectively; finally, in “E vs NE”, the boxes are populated with essential and nonessential genes, respectively.

Of the 31 available features, only features analyzed in the meta-study are automatically selected. By clicking on “Example: Essentiality loss”, a spreadsheet is downloaded containing values for the feature essentiality loss, which summarizes for each essential gene the number of species (evaluating *Candida albicans*, *Schizosaccharomyces pombe*, *Caenorhabditis elegans*, and the HAP1 and KBM7 human cell lines) with an absent, duplicated, or 1:1 nonessential ortholog [4]. Once the file is downloaded, it can be added as a user-defined feature by selecting it after clicking on “Upload file.”

By clicking on “Calculate”, Qarles performs the enrichment analyses for the selected features and generates the corresponding plots (see the “Materials and methods” section and Fig. 1). The resulting enrichments reveal very specific features for each gene set, and demonstrate that dispensable essential genes display features that lie between those of core essential and nonessential genes, challenging the classical binary classification of genes based on their essentiality. For instance, conservation is higher for the background genes in the “Dispensable vs core” comparison, but higher for the hit genes in “Dispensable vs NE” (Fig. 1D), which means that dispensable essential genes are less conserved than core essential genes, but more than nonessential genes, respectively. Additionally, dispensable essential genes also tend to have significantly lower coexpression degree and PPI degree, and higher gene copy number than core essential genes, as previously found [42]. Details on specific features can be found in the individual plots at the bottom, and by clicking on the “Interactive plots” button on the right, which allows the identification of outliers. Additionally, the specific relationship found between dispensable essential genes and conservation motivated downstream analyses across a panel of *S. cerevisiae* strains and on the phylogenetic properties in other species [42], which highlights how gene set characterization can drive hypothesis generation.

By clicking on “Predict hit genes (Dispensable vs core)”, Qarles automatically trains a random forest for the prediction of dispensable essential genes (i.e. the hit genes) by using the selected features. A dialog box reports the performance of the predictor and the number of genes used for training (genes with NA values in any of the features are not considered). The embedded table lists background and untested genes sorted by their prediction score. These predictions for untested genes can be validated against a list of dispensable essential genes reported in other studies and not used for training [42]. Out of the top and bottom 20 predictions, 7 and 0 are present in the validation set (*P* < .05), respectively, showing the relevance of the predictions generated by Qarles, which are completely automated and do not require any technical knowledge by the user. This performance is in line with predictions previously experimentally validated [4]. Note that small changes in the predictions across runs are to be expected since training generates a different random forest model each time.

In the example labeled “Compartments”, genes are grouped by their localization in the cell [43]. The resulting dotplot nicely summarizes the different properties of genes localizing at the nucleus, the cytoplasm, the mitochondrion, and the nucleolus, particularly in essentiality, being a transcription factor, or localizing at a membrane. Additionally, genes not found in any compartment result in very distinct enrichments and are more likely to be uncharacterized. Importantly, since five gene set comparisons are performed across all 31 features, this example provides an upper bound of the computational time needed to run Qarles.

The example “With orthologs” presents a simple case in which the “Hit list” and “Background list” boxes include genes with and without a human ortholog, respectively [35]. All 31 features are selected for analysis, and the resulting plots show how genes with a human ortholog are more likely to be essential and code for protein complex members, and are less likely to be uncharacterized, among many other significantly enriched features.

## Conclusion

Qarles is a free web server available at https://qarles.org that quickly characterizes large sets of *S. cerevisiae* genes. It provides reliable statistics and publication-ready figures, including several gene set comparisons across multiple features in a single plot. Information on the features is available on the website and the supplementary material, which is essential for the proper biological interpretation of the results by the user. Additionally, new hits can be predicted by a random forest trained on the selected features and gene lists. Altogether, with its unique functionality and user-friendly interface, Qarles provides an effective resource for the yeast community.

## Supplementary Material

lqaf030_Supplemental_File

## Data Availability

Qarles is freely available at https://qarles.org.
