# Supplementary material for: Qarles: a web server for the quick characterization of large sets of genes
Source: NAR Genom Bioinform. 2025 Mar 29;7(2):lqaf030. doi: 10.1093/nargab/lqaf030 (PMC11954521; doi:10.1093/nargab/lqaf030)
Supplement: lqaf030_Supplemental_File [file lqaf030_supplemental_file.docx]

| **Feature** | **Description** |
| --- | --- |
| Cocomplex degree | Total number of partners in the protein complexes in which the gene product is found [(1)](https://paperpile.com/c/H0ZuHz/ZeJE). Complex data downloaded from the Complex Portal (https://ftp.ebi.ac.uk/pub/databases/intact/complex/current/complextab/559292.tsv) on Dec 4th 2024. |
| Coexpression degree | Number of genes with a similar expression profile (i.e., MEFIT scores > 1) [(2)](https://paperpile.com/c/H0ZuHz/fJE9). MEFIT scores provided by authors. |
| Conservation (broad) | Number of distant species in which the gene is conserved [(3)](https://paperpile.com/c/H0ZuHz/FNqL) (additional file 3). |
| Conservation (yeasts) | Number of yeast species in which the gene is conserved [(3)](https://paperpile.com/c/H0ZuHz/FNqL) (additional file 3). |
| Degradation rate | Protein degradation rate [(4)](https://paperpile.com/c/H0ZuHz/NcZq) (table S1). |
| dN/dS | Ratio of non-synonymous to synonymous substitutions (dN/dS) to quantify sequence evolution [(3)](https://paperpile.com/c/H0ZuHz/FNqL) (additional file 3). |
| Expression variance (e) | Expression variance under different environmental conditions [(5)](https://paperpile.com/c/H0ZuHz/6PnW), calculated as previously described [(6)](https://paperpile.com/c/H0ZuHz/ms4D). |
| Expression variance (g) | Expression variance in different genetic backgrounds [(7)](https://paperpile.com/c/H0ZuHz/PGBv), calculated as previously described [(6)](https://paperpile.com/c/H0ZuHz/ms4D). |
| Gene copy number | Number of paralogs [(3)](https://paperpile.com/c/H0ZuHz/FNqL) (additional file 3). |
| mRNA halflife | mRNA halflife in CSM-lowURA media [(8)](https://paperpile.com/c/H0ZuHz/3P2h). Average across the two replicates found in supplemental file elife-32536-fig1-data2-v4.txt. |
| Multifunctionality | Number of GO SLIM biological process annotations [(9)](https://paperpile.com/c/H0ZuHz/dX6e). Annotations downloaded from the SGD (http://sgd-archive.yeastgenome.org/curation/literature/go_slim_mapping.tab) on Dec 4th 2024. |
| Number of complexes | Number of protein complexes in which the gene product is found [(1)](https://paperpile.com/c/H0ZuHz/ZeJE). Complex data downloaded from the Complex Portal (https://ftp.ebi.ac.uk/pub/databases/intact/complex/current/complextab/559292.tsv) on Dec 4th 2024. |
| Number of domains | Number of structural domains identiﬁed by Pfam 37.0 [(10)](https://paperpile.com/c/H0ZuHz/Z7D5). Families, repeats, motifs, and coiled-coils are not considered, and multiple instances of the same domain are only counted once. Domain data downloaded from https://ftp.ebi.ac.uk/pub/databases/Pfam/releases/Pfam37.0/proteomes/559292.tsv.gz on Dec 4th 2024. |
| PPI degree | Number of protein–protein interactions [(3)](https://paperpile.com/c/H0ZuHz/FNqL) (additional file 3). |
| Protein abundance | Median protein abundance in molecules per cell across 21 studies [(11)](https://paperpile.com/c/H0ZuHz/2o0d) (median value in Table S4). |
| Protein disorder | Fraction of structurally disordered residues calculated by VLS2b [(12)](https://paperpile.com/c/H0ZuHz/xTSt). Disorder data downloaded from d2p2.pro [(13)](https://paperpile.com/c/H0ZuHz/NQgb) on Dec 4th 2024 using Saccharomyces cerevisiae 63_3 as genome query. |
| Protein halflife | Protein halflife in minutes [(4)](https://paperpile.com/c/H0ZuHz/NcZq) (table S1). |
| Protein length | Number of residues in the protein [(14)](https://paperpile.com/c/H0ZuHz/Rbuq). Protein sequences downloaded from SGD (http://sgd-archive.yeastgenome.org/sequence/S288C_reference/orf_protein/orf_trans_all.fasta.gz) on Dec 4th 2024. |
| Single-mutant fitness | Single-mutant fitness for non-essential genes under standard conditions [(6)](https://paperpile.com/c/H0ZuHz/ms4D). Retrieved from Data file S1 (table strain_ids_and_single_mutant_fitness.xlsx), downloaded from https://boonelab.ccbr.utoronto.ca/supplement/costanzo2016/. |
| Transcript count | Transcript count [(15)](https://paperpile.com/c/H0ZuHz/Jm2Q) (Table S1). |
| Has a gene duplicate | Whether the gene has any duplicate [(16)](https://paperpile.com/c/H0ZuHz/4vYP). Paralog pairs downloaded from AllianceMine (Templates -> All S. cerevisiae Paralogs, https://www.alliancegenome.org/alliancemine/service/template/results?name=Gene_YeastParalogs&format=tab) on Dec 4th 2024. |
| Has a structural domain | Whether the gene codes for a protein with a structural domain [(10)](https://paperpile.com/c/H0ZuHz/Z7D5). Families, repeats, motifs, and coiled-coils are not considered. Domain data from Pfam 37.0 downloaded from https://ftp.ebi.ac.uk/pub/databases/Pfam/releases/Pfam37.0/proteomes/559292.tsv.gz on Dec 4th 2024. |
| Increases lifespan | Whether deletion of the gene increases lifespan [(17)](https://paperpile.com/c/H0ZuHz/EBmv) (Table S1 and S2). |
| Is at a membrane | Whether the gene codes for a protein localizing at a membrane [(18)](https://paperpile.com/c/H0ZuHz/WKcT). List of membrane proteins retrieved from Table S2 with status equal to 1. ANNOTATED MEMBRANE or 2. PUTATIVE MEMBRANE. |
| Is essential | Whether the gene is essential for viability in standard conditions [(19)](https://paperpile.com/c/H0ZuHz/QPLO) (Table S13). |
| Is in a protein complex | Whether the gene codes for a protein complex member [(1)](https://paperpile.com/c/H0ZuHz/ZeJE). Complex data downloaded from the Complex Portal (https://ftp.ebi.ac.uk/pub/databases/intact/complex/current/complextab/559292.tsv) on Dec 4th 2024. |
| Is a transcription factor | Whether the gene codes for a transcription factor [(20)](https://paperpile.com/c/H0ZuHz/Pbb8). Data provided by the YEASTRACT team (http://www.yeastract.com/) on Dec 9th 2024. |
| Is uncharacterized | Whether the gene has an unknown function [(16)](https://paperpile.com/c/H0ZuHz/4vYP). Data downloaded from AllianceMine (Lists -> Uncharacterized_ORFs) on Dec 4th 2024. |
| PTM: acetylation | Whether the gene has been previously reported as acetylated in the literature [(16)](https://paperpile.com/c/H0ZuHz/4vYP). Post-translational modifications downloaded from AllianceMine (Templates -> Post-translational modifications, https://www.alliancegenome.org/alliancemine/service/template/results?name=Literature_ProteinMods&format=tab) on Dec 4th 2024. |
| PTM: ubiquitination | Whether the gene has been previously reported as ubiquitinated in the literature [(16)](https://paperpile.com/c/H0ZuHz/4vYP). Post-translational modifications downloaded from AllianceMine (Templates -> Post-translational modifications, https://www.alliancegenome.org/alliancemine/service/template/results?name=Literature_ProteinMods&format=tab) on Dec 4th 2024. |
| PTM: phosphorylation | Whether the gene has been previously reported as phosphorylated in the literature [(16)](https://paperpile.com/c/H0ZuHz/4vYP). Post-translational modifications downloaded from AllianceMine (Templates -> Post-translational modifications, https://www.alliancegenome.org/alliancemine/service/template/results?name=Literature_ProteinMods&format=tab) on Dec 4th 2024. |

**Table S1. List of features in Qarles.** *Feature*: name of the feature. *Description*: description of the feature, including the publication from which the data was derived, and the supplementary data or download link if needed.

**REFERENCES**

[1. Meldal,B.H.M., Pons,C., Perfetto,L., Del-Toro,N., Wong,E., Aloy,P., Hermjakob,H., Orchard,S. and Porras,P. (2021) Analysing the yeast complexome-the Complex Portal rising to the challenge. *Nucleic Acids Res.*, **49**, 3156–3167.](http://paperpile.com/b/H0ZuHz/ZeJE)

[2. Huttenhower,C., Hibbs,M., Myers,C. and Troyanskaya,O.G. (2006) A scalable method for integration and functional analysis of multiple microarray datasets. *Bioinformatics*, **22**, 2890–2897.](http://paperpile.com/b/H0ZuHz/fJE9)

[3. Koch,E.N., Costanzo,M., Bellay,J., Deshpande,R., Chatfield-Reed,K., Chua,G., D’Urso,G., Andrews,B.J., Boone,C. and Myers,C.L. (2012) Conserved rules govern genetic interaction degree across species. *Genome Biol.*, **13**, R57.](http://paperpile.com/b/H0ZuHz/FNqL)

[4. Christiano,R., Nagaraj,N., Fröhlich,F. and Walther,T.C. (2014) Global proteome turnover analyses of the Yeasts S. cerevisiae and S. pombe. *Cell Rep.*, **9**, 1959–1965.](http://paperpile.com/b/H0ZuHz/NcZq)

[5. Gasch,A.P., Spellman,P.T., Kao,C.M., Carmel-Harel,O., Eisen,M.B., Storz,G., Botstein,D. and Brown,P.O. (2000) Genomic expression programs in the response of yeast cells to environmental changes. *Mol. Biol. Cell*, **11**, 4241–4257.](http://paperpile.com/b/H0ZuHz/6PnW)

[6. Costanzo,M., VanderSluis,B., Koch,E.N., Baryshnikova,A., Pons,C., Tan,G., Wang,W., Usaj,M., Hanchard,J., Lee,S.D., *et al.* (2016) A global genetic interaction network maps a wiring diagram of cellular function. *Science*, **353**](http://paperpile.com/b/H0ZuHz/ms4D)[, aaf1420](http://paperpile.com/b/m1fZIo/Me7T5).

[7. Skelly,D.A., Merrihew,G.E., Riffle,M., Connelly,C.F., Kerr,E.O., Johansson,M., Jaschob,D., Graczyk,B., Shulman,N.J., Wakefield,J., *et al.* (2013) Integrative phenomics reveals insight into the structure of phenotypic diversity in budding yeast. *Genome Res.*, **23**, 1496–1504.](http://paperpile.com/b/H0ZuHz/PGBv)

[8. Chan,L.Y., Mugler,C.F., Heinrich,S., Vallotton,P. and Weis,K. (2018) Non-invasive measurement of mRNA decay reveals translation initiation as the major determinant of mRNA stability. *Elife*, **7**](http://paperpile.com/b/H0ZuHz/3P2h)[, e32536.](http://paperpile.com/b/m1fZIo/w7vp3)

[9. Ashburner,M., Ball,C.A., Blake,J.A., Botstein,D., Butler,H., Cherry,J.M., Davis,A.P., Dolinski,K., Dwight,S.S., Eppig,J.T., *et al.* (2000) Gene ontology: tool for the unification of biology. The Gene Ontology Consortium. *Nat. Genet.*, **25**, 25–29.](http://paperpile.com/b/H0ZuHz/dX6e)

[10. Mistry,J., Chuguransky,S., Williams,L., Qureshi,M., Salazar,G.A., Sonnhammer,E.L.L., Tosatto,S.C.E., Paladin,L., Raj,S., Richardson,L.J., *et al.* (2021) Pfam: The protein families database in 2021. *Nucleic Acids Res.*, **49**, D412–D419.](http://paperpile.com/b/H0ZuHz/Z7D5)

[11. Ho,B., Baryshnikova,A. and Brown,G.W. (2018) Unification of Protein Abundance Datasets Yields a Quantitative Saccharomyces cerevisiae Proteome. *Cell Syst*, **6**, 192–205.e3.](http://paperpile.com/b/H0ZuHz/2o0d)

[12. Peng,K., Radivojac,P., Vucetic,S., Dunker,A.K. and Obradovic,Z. (2006) Length-dependent prediction of protein intrinsic disorder. *BMC Bioinformatics*, **7**, 208.](http://paperpile.com/b/H0ZuHz/xTSt)

[13. Oates,M.E., Romero,P., Ishida,T., Ghalwash,M., Mizianty,M.J., Xue,B., Dosztányi,Z., Uversky,V.N., Obradovic,Z., Kurgan,L., *et al.* (2013) D^2^P^2^: database of disordered protein predictions. *Nucleic Acids Res.*, **41**, D508–16.](http://paperpile.com/b/H0ZuHz/NQgb)

[14. Wong,E.D., Miyasato,S.R., Aleksander,S., Karra,K., Nash,R.S., Skrzypek,M.S., Weng,S., Engel,S.R. and Cherry,J.M. (2023) Saccharomyces genome database update: server architecture, pan-genome nomenclature, and external resources. *Genetics*, **224**](http://paperpile.com/b/H0ZuHz/Rbuq)[, iyac191.](http://paperpile.com/b/m1fZIo/uz3RV)

[15. Lipson,D., Raz,T., Kieu,A., Jones,D.R., Giladi,E., Thayer,E., Thompson,J.F., Letovsky,S., Milos,P. and Causey,M. (2009) Quantification of the yeast transcriptome by single-molecule sequencing. *Nat. Biotechnol.*, **27**, 652–658.](http://paperpile.com/b/H0ZuHz/Jm2Q)

[16. Engel,S.R., Wong,E.D., Nash,R.S., Aleksander,S., Alexander,M., Douglass,E., Karra,K., Miyasato,S.R., Simison,M., Skrzypek,M.S., *et al.* (2022) New data and collaborations at the Saccharomyces Genome Database: updated reference genome, alleles, and the Alliance of Genome Resources. *Genetics*, **220**](http://paperpile.com/b/H0ZuHz/4vYP)[, iyab224.](http://paperpile.com/b/m1fZIo/aNqCE)

[17. McCormick,M.A., Delaney,J.R., Tsuchiya,M., Tsuchiyama,S., Shemorry,A., Sim,S., Chou,A.C.-Z., Ahmed,U., Carr,D., Murakami,C.J., *et al.* (2015) A comprehensive analysis of replicative lifespan in 4,698 single-gene deletion strains uncovers conserved mechanisms of aging. *Cell Metab.*, **22**, 895–906.](http://paperpile.com/b/H0ZuHz/EBmv)

[18. Babu,M., Vlasblom,J., Pu,S., Guo,X., Graham,C., Bean,B.D.M., Burston,H.E., Vizeacoumar,F.J., Snider,J., Phanse,S., *et al.* (2012) Interaction landscape of membrane-protein complexes in Saccharomyces cerevisiae. *Nature*, **489**, 585–589.](http://paperpile.com/b/H0ZuHz/WKcT)

[19. van Leeuwen,J., Pons,C., Tan,G., Wang,J.Z., Hou,J., Weile,J., Gebbia,M., Liang,W., Shuteriqi,E., Li,Z., *et al.* (2020) Systematic analysis of bypass suppression of essential genes. *Mol. Syst. Biol.*, **16**, e9828.](http://paperpile.com/b/H0ZuHz/QPLO)

[20. Monteiro,P.T., Oliveira,J., Pais,P., Antunes,M., Palma,M., Cavalheiro,M., Galocha,M., Godinho,C.P., Martins,L.C., Bourbon,N., *et al.* (2020) YEASTRACT+: a portal for cross-species comparative genomics of transcription regulation in yeasts. *Nucleic Acids Res.*, **48**, D642–D649.](http://paperpile.com/b/H0ZuHz/Pbb8)
